# Cerebral Blood Flow Velocity Modulation and Clinical Efficacy of Acupuncture for Posterior Circulation Infarction Vertigo: A Systematic Review and Meta-Analysis

**DOI:** 10.1155/2022/3740856

**Published:** 2022-06-28

**Authors:** Boxuan Li, Qi Zhao, Yuzheng Du, Xiayu Li, Zefang Li, Xianggang Meng, Chen Li, Zhihong Meng, Junjie Chen, Chaoda Liu, Beidi Cao, Shihao Chi

**Affiliations:** ^1^First Teaching Hospital of Tianjin University of Traditional Chinese Medicine, Changling Road, No. 88, Xiqing District, Tianjin, China; ^2^National Clinical Research Center for Chinese Medicine Acupuncture and Moxibustion, Changling Road, No. 88, Xiqing District, Tianjin, China; ^3^Tianjin University of Traditional Chinese Medicine, Beihuanan Road, Jinghai District, Tianjin, China

## Abstract

**Background:**

Vertigo is a cardinal symptom of posterior circulation infarction (POCI). Acupuncture is demonstrated to have a beneficial effect on posterior circulation infarction vertigo (PCIV). However, the mechanism of acupuncture therapy is not clarified. This study aims to assess the cerebral blood flow velocity modulation and clinical efficacy of acupuncture for PCIV patients.

**Methods:**

We conducted this systematic review for clinical randomized controlled trials (RCTs) regarding acupuncture on PCIV. The study duration was from September 2020 to September 2021. We searched the PubMed, EMBASE, Cochrane Library, Web of Science, Chinese Biomedical Literature Database (CBM), China National Knowledge Infrastructure (CNKI), Wanfang Database, and VIP. The publication date was set from inception to August 31, 2020. Based on the inclusion and exclusion criteria, two researchers independently screened literature and extracted data including basic study information, intervention details, outcome details, and adverse events. Outcome measures included the blood flow velocities of vertebrobasilar arteries and the Clinical Effective Rate of posterior circulation infarction vertigo. Pooled data were presented as standardized mean differences (SMDs) and relative risks (RR), with 95% confidence intervals (CIs). The meta-analysis was conducted using Review Manager software version 5.3.0.

**Results:**

A total of 20 eligible RCTs (1541 participants) were included in this review, which compared acupuncture therapy (1 RCT) or acupuncture combined with pharmaceutical therapy (19 RCTs) to pharmaceutical therapy in patients with posterior circulation infarction vertigo. 7 studies assessed the blood flow velocities of the basilar artery examined by Transcranial Doppler (TCD), 8 studies assessed the bilateral vertebral arteries, and 13 studies evaluated the Clinical Effective Rate of posterior circulation infarction vertigo. Meta-analysis results showed that blood flow velocities of the basilar artery (SMD = 0.58, 95% CI = 0.40–0.76; *P* < 0.05), left vertebral artery (SMD = 0.48, 95% CI = 0.22–0.73; *P* < 0.05), and right vertebral artery (SMD = 0.44, 95% CI = 0.19–0.69; *P* < 0.05) were significantly higher in the acupuncture group compared with the control group. Clinical Effective Rate (RR = 1.22, 95% CI = 1.15–1.29; *P*  =  0.792) was significantly better in the acupuncture group compared with the control group.

**Conclusions:**

This study shows that acupuncture therapy is useful in improving the blood flow velocity of vertebrobasilar arteries and Clinical Effective Rate in patients with posterior circulation infarction vertigo. However, double-blind, sham-controlled trials with large sample sizes are required to support our conclusions.

## 1. Introduction

Posterior circulation infarction is the infarction of the vertebrobasilar arterial system that accounts for 20%–25% of ischemic stroke [1, 2]. Compared with the diagnosis of anterior circulation infarction, clinicians face more difficulties in the early diagnosis of POCI [3]. Hence, the importance of accurately assessing ischemic stroke in the posterior circulation is highlighted, and the utility of symptoms is emphasized [4]. Vertigo is a common complaint in the clinic with a high risk of recurrence [5, 6]. According to the epidemiological survey, vertigo is one of the most common symptoms of POCI, occurring in 47%–75% of posterior circulation stroke patients [7, 8]. An ischemic stroke population-based study demonstrated that vertigo attacks emerged more frequently in the vertebrobasilar system compared with the anterior system, especially during the preceding two days [9]. The rising prevalence of vertigo increases the burden of healthcare costs [10]. Meanwhile, cerebrovascular events lead to plenty of disabled patients who need permanent treatment and caregiving. It is estimated that stroke accounts for 2%–4% of global healthcare costs. The cost is increasing due to the burgeoning elderly population [11].

Vertigo is a subtype of dizziness, defined as the sensation of self-motion when no self-motion is occurring, or the sensation of distorted self-motion in otherwise normal head movement [12, 13]. For vertigo caused by infarction of the brain stem, cerebellum, or labyrinth due to vertebrobasilar arterial ischemia, the symptom might last for weeks to months [14]. Long time and severe vertigo disrupt patients' normal activities, affecting the daily life of about a fifth of patients over 60 years old [15]. Apart from treatments that focus on vertigo, management of POCI is necessary [2, 16]. Nevertheless, the current medical management strategy for POCI parallels that of anterior circulation infarction. Thus, specific therapies that focus on POCI and PCIV are needed [17]. Acupuncture, as a complementary and alternative therapy, provides a new solution for PCIV patients.

Acupuncture has been practiced for more than 2000 years and has acquired abundant experience in poststroke rehabilitation [18, 19]. With the advantage of effectiveness, acupuncture has been applied in more than 183 countries and areas all over the world [20, 21]. Meanwhile, the safety of acupuncture has been approved by quantitative evidence. The adverse events related to acupuncture are usually mild, and most of them can be avoided through standardizing clinical practices [22, 23]. With the rapidly growing research on the clinical application of acupuncture, the evidence of acupuncture's therapeutic effects on neurological disease has increased [24]. Clinical studies have proved the superior effects of acupuncture on ameliorating vertigo severity and improving the vertebrobasilar blood flow velocity in PCIV patients [25, 26]. Acupuncture has also shown powerful effects in alleviating patients' activity functions and clinical symptoms, especially in improving score of Activity of Daily Living (ADL) scale, and reducing the duration of vertigo attack [27, 28]. Although clinical studies have demonstrated the safety and effectiveness of acupuncture for PCIV [29, 30], evidence-based research in this area is lacking. Meanwhile, the cerebral blood flow modulation effect of acupuncture needs a quantitative assessment. Hence, the objective of this study is to evaluate the cerebral blood flow modulation effect and clinical efficacy of acupuncture for PCIV patients through meta-analysis. This study might provide a basis for further exploration in the future. The results will suggest up-to-date evidence for clinicians and an alternative option for patients.

## 2. Material and Method

Following the Preferred Reporting Items for Systematic Reviews and Meta-Analyses guidelines, this study protocol was registered in PROSPERO (Registration number: CRD42020199622) and INPLASY (Registration number: INPLASY202070116) and was published on September 11, 2020 [31].

### 2.1. Eligibility Criteria

Types of study are as follows: randomized controlled trials that explored the cerebral blood flow velocity modulation effects and clinical efficacy of acupuncture for PCIV were included. Quasi-RCTs, animal studies, case reports, and reviews were excluded.

Types of patients are as follows: patients that were clinically diagnosed with PCIV. According to the WHO diagnostic criteria for PCIV [32], patients must have dizziness or vertigo that may be accompanied by neurological deficiency. The imaging evidence of posterior circulation infarction, magnetic resonance imaging, or computed tomography was needed. There were no limitations on patients' gender, age, race, or the course of the disease.

Types of interventions are as follows: patients in the experimental group received acupuncture therapy alone, including MA, EA, and scalp acupuncture, or acupuncture therapy with pharmaceutical therapy. Patients in the control group received pharmaceutical therapy, placebo treatment such as sham acupuncture, or no treatment. Studies that focused on comparisons between different acupuncture methods or different acupoints were excluded.

Types of outcomes are as follows:

1. Cerebral posterior circulation conditions, such as the vertebrobasilar blood flow velocity value that was examined by TCD.
2. Clinical Effective Rate, as defined by Guiding Principles for Clinical Study of New Chinese Medicines and Criteria for the Diagnosis and Treatment of Traditional Chinese Medicine Syndrome [33, 34]. The Clinical Effective Rate is the proportion of participants whose vertigo symptoms improved by at least 30% after treatment to the total number of participants in the control or acupuncture group.

### 2.2. Search Strategy

The systematic literature search was conducted in the following databases: PubMed, EMBASE, Cochrane Library, Web of Science, CBM, CNKI, Wanfang Database, and VIP from inception to August 31, 2020, without language restrictions. The keywords for literature retrieval were “vertigo,” “posterior circulation,” “vertebrobasilar,” “stroke,” “cerebral infarction,” “acupuncture,” “electroacupuncture,” and “needle.” The search terms and search strategy are presented in Supplementary Table S1.

### 2.3. Study Selection and Data Extraction

Two reviewers (BXL and XYL) independently selected studies by reading titles, abstracts, and full articles and then extracted data using the predefined criteria. The following data were extracted from the included studies: (1) basic information of the study including the first author's last name, year of publication, study design, participant's age, participant gender, outcome index, and adverse events; (2) intervention details including intervention type, intervention course, acupuncture type, main acupoints, reinforcing and reducing acupuncture manipulation, acupuncture retention time; (3) primary outcome value, secondary outcome value before and after intervention; (4) adverse events.

### 2.4. Quality Assessment

Two reviewers (BXL and ZFL) assessed the bias risk of the included trials according to the updated Cochrane Risk of Bias 2 (RoB 2) tool [35]. Each trial was classified as high risk, low risk, or some concerns in five domains: randomization process, deviations from intended interventions, missing outcome data, measurement of the outcome, and selection of the reported result. An overall trial bias risk was graded based on the above 5 aspects. Using IBM SPSS Statistics version 21 (IBM SPSS Inc., Chicago, USA), we calculated the Cohen's kappa coefficient (*κ*) to evaluate the agreement between the two investigators (BXL and ZFL). Agreement was judged as poor if the *κ* ≤0.2, fair if 0.2 < *κ* ≤ 0.4, moderate if 0.4 < *κ* ≤ 0.6, substantial if 0.6 < *κ* ≤ 0.8, and perfect if *κ* >0.8.

### 2.5. Statistical Analysis

To conduct statistical analysis, we used Review Manager (RevMan) software version 5.3.0 (Cochrane Central Executive Team, United Kingdom) as recommended by Cochrane Collaboration. The standard mean difference was used to represent continuous data, and the relative risks were used to represent dichotomous data. Results were reported with 95% confidence intervals, and *P* < 0.05 was considered a significant statistical effect.

For heterogeneity analysis, an *I*^2^ test was performed. A random-effects model was conducted if significant heterogeneity was found as *I*^2^ ＞ 50%. Otherwise, a fixed-effects model was used. Subgroup analysis was conducted for heterogeneity that arose from interventions, treatment duration, acupoints, and outcome indicators.

## 3. Results

### 3.1. Searching Results

**Figure 1:**
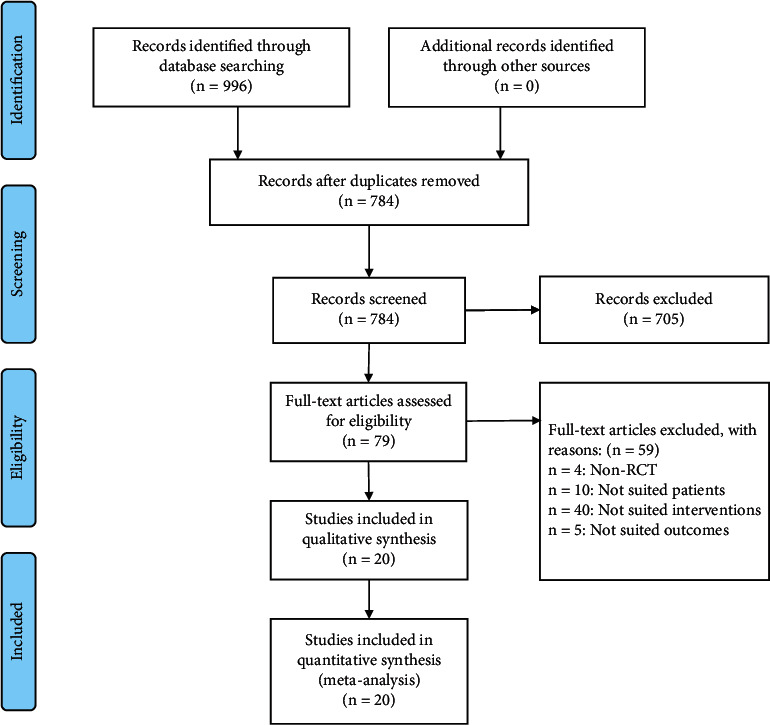
Flow diagram of study selections.

A total of 996 potentially relevant articles were searched from 8 databases (Supplementary Table S2), and 784 remained after removing duplicates. 705 articles were excluded based on the titles and abstracts. Then, we reviewed the remaining 79 full-text articles. Eventually, 20 articles were included in the analysis (Figure 1).

### 3.2. Study Characteristics

Study characteristics including author names, publication years, study design, sample size, participant age, participant gender, intervention types, intervention course, and outcome indicators are summarized in Table 1. Characteristics of acupuncture including main acupoints, acupuncture types, reinforcing and reducing acupuncture manipulation, and needle retention time are summarized in Table 2.

Among the included 20 articles, 19 articles were in Chinese, and 1 was in English. A total of 1541 participants were included in this meta-analysis, 797 in the intervention group and 744 in the control group. For intervention types, 7 studies compared acupuncture plus specific medicine to specific medicine (Flunarizine hydrochloride, Betastatin, Aspirin, Danhong injection, Pushen capsule, Alprostadil injection, and Gastrodin injection); 6 studies compared acupuncture plus conventional treatment to conventional treatment; 6 studies compared acupuncture plus conventional treatment to conventional treatment plus specific medicine (Flunarizine hydrochloride, Nimodipine, and Vinpocetine injection); and 1 study compared acupuncture therapy to medicine treatment (Flunarizine hydrochloride). The conventional treatment consisted of pharmacological treatment for antiplatelet, antihypertensive, and blood sugar regulation. For the acupuncture type, 17 studies used manual acupuncture (MA), and 3 studies used manual acupuncture plus electroacupuncture (MA plus EA). For the acupoint selection, 33 main acupoints were used in the 20 studies, and GB 20 (Fengchi) appeared in 15 studies (75%), which was the most frequently used acupoint.

### 3.3. Methodological Evaluation

**Figure 2:**
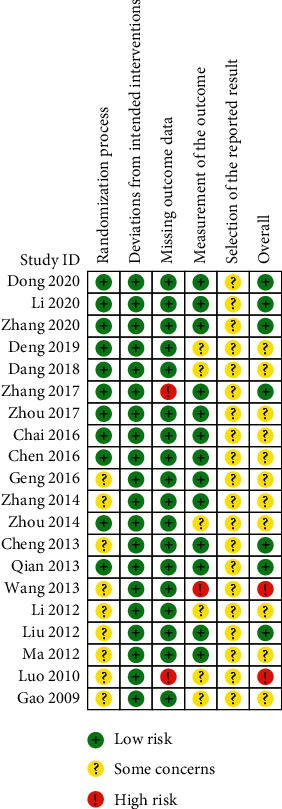
Risk of bias summary.

**Figure 3:**
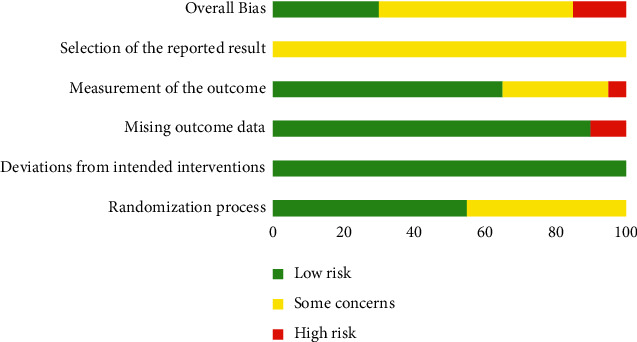
Risk of bias graph.

Using the RoB 2 and Cohen's kappa coefficient, two reviewers (BXL and ZFL) independently assessed the risk of bias of the included randomized controlled trials (Table 3, Figures 2 and 3). For the randomization process, 11 studies that mentioned specific methods (computer randomization or table of random numbers) were considered at low risk (55%). As for deviations from intended interventions, all studies were considered at low risk. For the missing outcome data evaluation, 2 studies that reported missing data were scored as high risk, and 18 studies were rated as low risk (90%). For the measurement of the outcome, 13 studies were at low risk (65%), 1 study was at high risk, and 6 studies with some concerns might have had potential bias due to the unblinding assessment. None of the 20 studies had published trial protocols or prespecified analysis plans, so they were all considered some concerns. Overall, 6 studies were judged as low risk (30%), 11 studies were considered some concerns, and 3 studies were considered at high risk. Cohen's *κ* values of deviations from intended interventions, measurement of the outcome, selection of the reported result, and overall bias were 1, which suggested a perfect agreement. Cohen's *κ* of randomization process and missing outcome data were 0.688, which represented a substantial agreement.

### 3.4. Meta-Analysis Results

Among the included 20 studies, meta-analysis was conducted with the outcomes of vertebrobasilar blood flow velocity and the Clinical Effectiveness Rate, including 9 RCTs reporting the blood flow velocity of basilar artery (BA), 10 RCTs assessing the blood flow velocity of the left vertebral artery (LVA) and the right vertebral artery (RVA) separately, and 19 RCTs evaluating the Clinical Effectiveness Rate. Apart from that, 1 study reported the Dizziness Handicap Inventory (DHI), 2 studies reported the ADL, and 3 studies evaluated the Traditional Chinese Medicine Vertigo Symptom Scale. Given the small number of studies with DHI, ADL, and Traditional Chinese Medicine Vertigo Symptom Scale, we did not conduct meta-analysis of these RCTs.

Hypoperfusion is considered a crucial mechanism of ischemic stroke [56]. TCD has been used in monitoring hemodynamic changes for poststroke patients [57]. According to clinical practice and study outcomes, we analyzed the blood flow velocity of the basilar artery, left vertebral artery, and right vertebral artery.

#### 3.4.1. The Blood Flow Velocity of Basilar Artery

**Figure 4:**
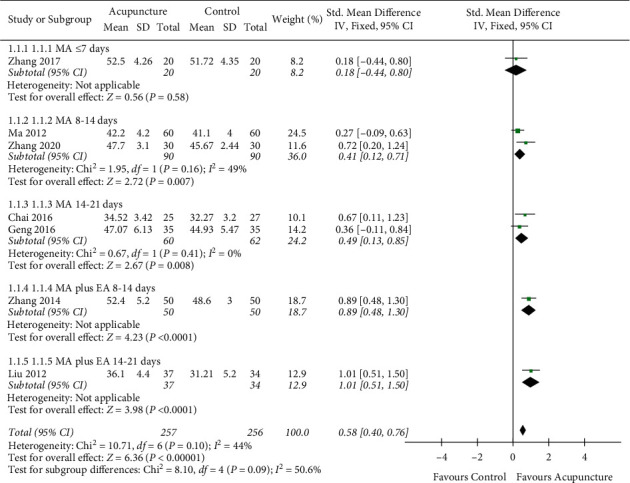
The blood flow velocity of basilar artery.

A total of 9 studies reported the blood flow velocity of BA. 2 studies were excluded due to the lack of quantified value [36] or the unbalanced baseline data [49]. Thus, 7 studies [38, 41, 43, 45, 46, 52, 53] with 513 participants were included in the analysis. The heterogeneity test showed a relatively low heterogeneity between groups (*I*^2^ = 44%, *P*=0.10). To find out the possible origins of heterogeneity, we conducted a sensitivity analysis. After reconsidering the baseline information and interventions, we found that acupuncture types with MA plus EA were different from studies with MA alone. Another possible reason was the intervention course, which varies from 7 days to 42 days. Acupuncture intervention course has been considered as a vital factor for stimulation dosage, and previous studies also revealed the dose-dependent effect [58, 59]. Therefore, we divided the 8 studies into 5 groups according to different acupuncture types and courses (Figure 4).

The results of the meta-analysis indicated a significant difference in favor of the acupuncture group (SMD = 0.58, 95% CI = 0.40–0.76; *P* < 0.05). In the subgroup analysis, groups with MA plus EA showed superior effects compared to MA groups. Within both MA groups and MA plus EA groups, the effects seemed to increase with the extension of the intervention course (Figure 4).

#### 3.4.2. The Blood Flow Velocity of Left Vertebral Artery

**Figure 5:**
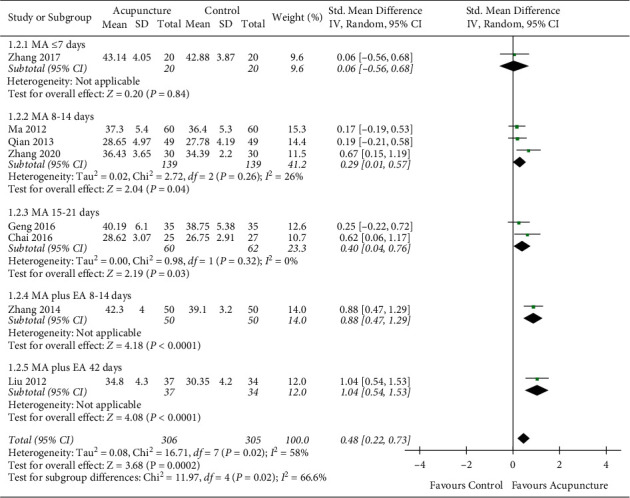
The blood flow velocity of left vertebral artery.

Among the included 20 studies, 10 studies reported the blood flow velocity of LVA and RVA separately. Two studies were excluded due to the lack of quantified value [36] and an unclear description of the left or right side being examined [37]. Eventually, 8 studies [38, 41, 43, 45, 46, 49, 52, 53] with 614 participants were included in the analysis of the blood flow velocity of LVA and RVA separately (Figure 5).

For the meta-analysis of the blood flow velocity of LVA, the heterogeneity test showed significant heterogeneity between groups (*I*^2^ = 58%, *P*=0.02). After conducting the sensitivity analysis, we divided the 8 studies into 5 groups according to different acupuncture types and courses. The meta-analysis implied a significant difference in favor of the acupuncture group (SMD = 0.48, 95% CI = 0.22–0.73; *P* < 0.05). Subgroup analysis showed that compared to all of the three MA groups, groups with MA plus EA had greater effects. The effects within both MA plus EA groups and MA groups presented a growing tendency with the extension of the intervention course (Figure 5).

#### 3.4.3. The Blood Flow Velocity of Right Vertebral Artery

**Figure 6:**
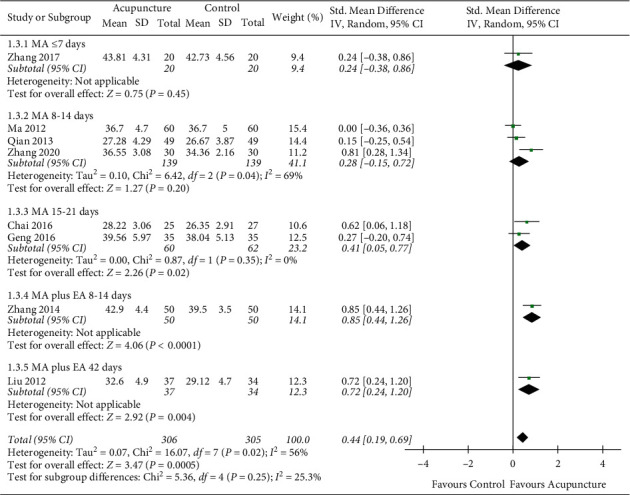
The blood flow velocity of right vertebral artery.

For the meta-analysis of the blood flow velocity of RVA, the heterogeneity test result showed significant heterogeneity between groups (Figure 6: *I*^2^ = 56%, *P*=0.02). After conducting the sensitivity analysis, 8 studies were divided into 5 groups according to different acupuncture types and courses. A significant difference in favor of the acupuncture group was indicated (SMD = 0.44, 95% CI = 0.19–0.69; *P* < 0.05). Subgroup analysis implied greater effects in MA plus EA groups compared to MA groups. Within both the MA plus EA groups and the MA groups, growing trends with the extension of the intervention course were presented (Figure 6).

#### 3.4.4. Clinical Effectiveness Rate

**Figure 7:**
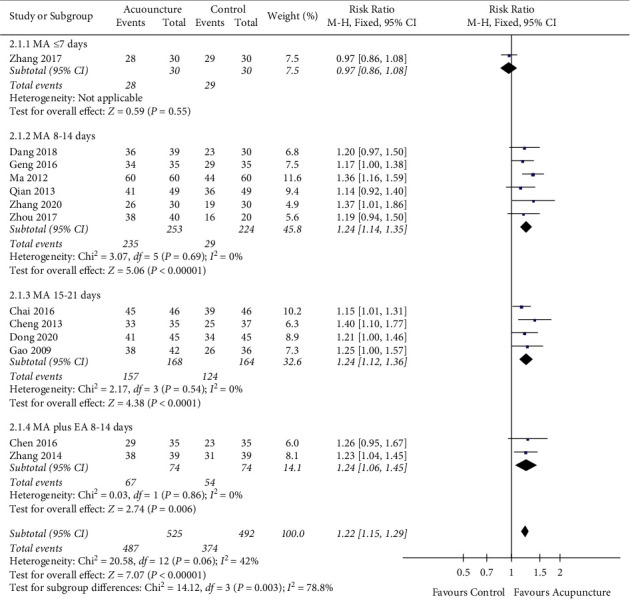
Clinical Effectiveness Rate.

Out of the included 20 studies, 19 [36, 38–55] studies reported the Clinical Effectiveness Rate. 6 studies were excluded as 3 studies did not mention specific evaluation criteria [39, 50, 51] and 3 studies [47, 52, 54] used inconsistent evaluation criteria. Eventually, 13 studies [36, 38, 40–46, 48, 49, 53, 55] with 1017 participants were included for meta-analysis. The heterogeneity test showed low heterogeneity between groups (Figure 7: *I*^2^ = 42%, *P*=0.06). After conducting the sensitivity analysis, we found that for acupuncture type, 2 studies used MA plus EA, and the other 11 studies used MA alone. Out of the 11 MA studies, one study used a 7-day acupuncture intervention course, while the other 10 studies varied from 10 days to 21 days. Therefore, we suspected the heterogeneity originating from the acupuncture intervention course. Subgroup analysis was conducted by dividing the 13 studies into 4 groups according to acupuncture type and course (Figure 7).

The result showed a significant difference in favor of the acupuncture group compared to the control group in clinical efficacy rate (RR = 1.22, 95% CI = 1.15–1.29; *P* < 0.05). Subgroup analysis indicated a weak effect in the group of MA for ≤ 7 days (RR = 0.97, 95% CI = 0.86–1.08) compared to the other three groups. There were minor differences in subgroup analysis between groups of 8–14-day MA (RR = 1.24, 95% CI = 1.14–1.35), 8–14-day MA plus EA (RR = 1.24, 95% CI = 1.06–1.45), and 15–21-day MA (RR = 1.24, 95% CI = 1.12–1.36).

#### 3.4.5. Safety

7 RCTs [39, 41, 46, 48, 52–54] reported the safety of acupuncture treatment. No adverse events caused by acupuncture were reported. Among them, 3 studies [39, 53, 54] reported no adverse events that were related to interventions during trials. One study [41] reported adverse reactions in the control group, including 4 participants with fatigue and 3 participants with sleepiness. Besides, 2 participants in the control group dropped out due to adverse reactions to the pharmacological treatment.

### 3.5. Publication Bias

**Figure 8:**
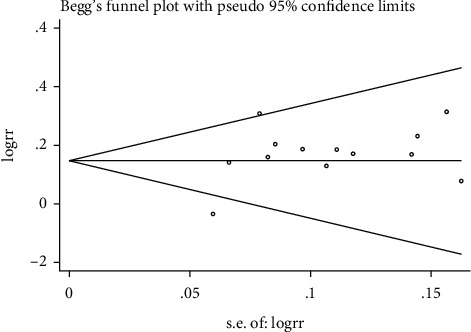
Begg's funnel plot.

**Figure 9:**
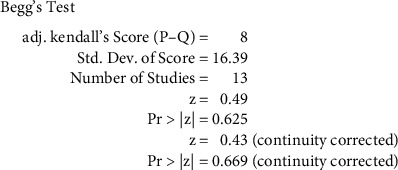
Begg's test.

**Figure 10:**
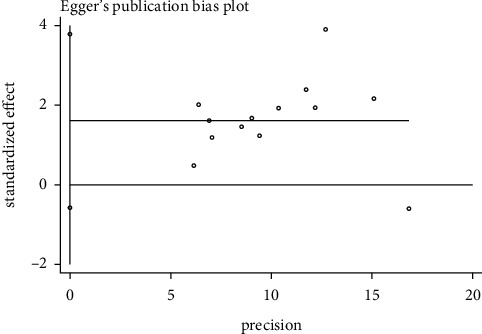
Egger's publication bias plot.

Publication bias was evaluated for studies with the clinical efficacy rate (13 studies). For studies with the blood flow velocity of LVA, RVA, and BA, publication bias was not evaluated since the study number was less than 10. We conducted the assessment using STATA software version 14 with Begg's test (Figures 8 and 9) and Egger's test methods (Figure 10, Table 4). The results did not show a significant difference in publication bias between the 13 studies (coefficient = 1.602403, 95% CI = −0.580–3.785; *P*=0.792).

## 4. Discussion

The posterior circulation system is also called the vertebrobasilar system, which is composed of the vertebral artery, basilar arteries, and their branches [2]. Compared to other types of vertigo, PCIV might be more dangerous, so it should be given more attention by clinicians and patients [60]. Current management of PCIV is consistent with stroke in general, lacking a specific therapy [61, 62]. Acupuncture's therapeutic effects have been proved in relieving vertigo symptoms and reducing neurological impairment [38]. However, the systematic evidence from clinical research is insufficient. Therefore, it is crucial to evaluate the therapeutic effect of acupuncture on PCIV patients.

As far as we know, this article first reported the systematic review of the clinical efficacy and blood flow velocity change for acupuncture interventions on PCIV. In previous studies, JX Chen reviewed the efficacy of acupuncture therapy for PCIV in 2018, focusing on 14 RCTs with an effectiveness rate of acupuncture treatment and medical treatment [63]. The results focused on the effectiveness rate only. Besides, the study mentioned low methodological quality in most of the included RCTs as well as obvious publication bias that originated from unreported negative results. In 2017, Hou et al. examined the efficacy of acupuncture therapy for cervical vertigo, analyzing 10 RCTs with an effectiveness rate and 3 RCTs with blood flow velocity differences between acupuncture treatment and medicine treatment [64]. The results showed that acupuncture might be more effective in relieving clinical symptoms and improving mean blood flow velocity, but they did not give a firm conclusion due to the low methodological quality. Compared to their reviews, this study used rigorous criteria to reduce the influence of other physical therapies. Furthermore, the included updated 20 RCTs with 1541 participants decreased the bias from a small sample size. Moreover, the analysis of clinical efficacy comprehensively reflected both symptoms and posterior circulation conditions of PCIV patients. Additionally, we conducted a subgroup analysis according to acupuncture stimulation dosage, which might help to reveal the acupuncture dose-effect.

In this review, a meta-analysis was conducted on acupuncture therapy for PCIV in vertebrobasilar blood flow velocity and Clinical Effectiveness Rate. The results of our research provided the following implications for clinical practice and further studies: Firstly, this study proved that compared to pharmaceutic therapy, acupuncture therapy alone or acupuncture combined with pharmaceutic therapy was more effective in the treatment of posterior circulation infarction vertigo. According to the meta-analysis results, acupuncture therapy showed superior effects on cerebral blood flow modulation and clinical syndrome reduction. These findings provide a basis for exploring the possible mechanism of acupuncture on PCIV.

In the mechanism studies revealing the physiological and biochemical basis behind the acupuncture therapy interventions over PCIV patients, the reconstruction of CBF has been demonstrated as a key process [65]. According to an animal experiment, a delayed CBF increase on the infarction border has been detected at 7–28 days after the infarct, which emphasized the significance of CBF reestablishment [66]. Acupuncture showed beneficial effects in improving hemodynamic indices of cerebral vessels, especially in enhancing the blood flow quantity of the vertebral artery [67]. Additionally, experimental researches conducted in our research center indicated acupuncture's effects on upregulating endothelial cell proliferation and increasing cerebral blood vessel number, which helps rebuild the CBF and save the neurological function [68]. Animal study has proved that acupuncture enhanced the blood flow volume of the vestibular nuclei area in PCIV rats [69]. Apart from cerebral blood flow modulation, the therapeutic mechanism behind acupuncture treatment is associated with multiple factors via complex metabolism pathways. In 2021, Meng's research found that acupuncture can improve the Glutamic Acid and Gamma-aminobutyric acid levels in PCIV rats, which lessens the excitability toxicity of ischemic stroke [70]. These findings validated the strength of acupuncture in improving the ischemic state via enhancing CBF. In this study, the meta-analysis results of the blood flow velocity changes confirmed the effect pathway of acupuncture on modulating cerebral blood flow. While most of the studies paid attention to the anterior circulation, our study focused on the posterior circulation. The results will provide new systematic evidence for further study.

Secondly, the subgroup analysis found some surprising results. In this study, the subgroups were identified based on acupuncture intervention course and acupuncture type, both of which were fundamental components of acupuncture stimulation does [71]. Subgroup analysis of the blood flow velocity in LVA, RVA, and BA showed that MA plus EA had better therapeutic effects than MA alone (Figures 4–6). The potential mechanism can be associated with acupuncture's dose-effect. Different acupuncture dosages will result in a great deal of variation between trials [72]. In this study, subgroup analysis showed that the acupuncture dosage was the primary cause of heterogeneity between groups. The result stressed the importance of acupuncture's dose-effect, which should be noted in further studies. In the case of cerebral disease, it has been demonstrated that different manipulation and stimulation amounts significantly influenced the therapeutic effect in enhancing cerebral blood flow in ischemic rats [73, 74]. Additionally, acupuncture's dose-effect is related to manipulation, retention time, needling depth, and other parameters [75, 76]. More well-designed trials are needed to explore the optimal dosage of acupuncture. The quantitative studies of acupuncture will not only seek a better dose-effect but also help to establish the standard of acupuncture application.

**Figure 11:**
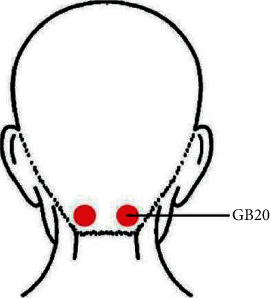
GB 20 (Fengchi).

Thirdly, by summarizing the acupoints for PCIV, this study found that the most frequently used acupoint in the 20 RCTs was GB 20 (Table 2). GB 20 is located in the posterior neck, inferior to the occipital bone, in the depression between the upper portion of the sternocleidomastoid and trapezius (Figure 11). The vertebral artery runs in the deep of GB 20 point, beneath the posterior atlantooccipital membrane [77]. According to Traditional Chinese Medicine theory, acupuncture has a therapeutic effect on diseases and disorders by regulating Qi and blood through the meridian system. It is recorded in TCM theory that PCIV is caused by the disorder of Qi and blood, and GB 20 has special effects on vertigo. Modern medical research indicates that acupuncture GB 20 can improve cerebral posterior circulation [78, 79]. Therefore, we highlight the use of GB 20 for PCIV in clinical practice.

This study also has some limitations. Firstly, due to the nature of acupuncture therapy and trial design, the participants and therapists in the 20 trials were unblinded to the treatments. Since the placebo effect might introduce a potential bias, an appropriate blinding procedure such as sham acupuncture is necessary. Though studies demonstrated the superiority of acupuncture's effect over sham acupuncture [80, 81], placebo effects were considerable [82]. To reveal the true effect of acupuncture and minimize potential bias, we strongly recommend the conduction of the blinding procedure. Blinding interventions such as blunt needles without penetration and superficial needling are applied in research. Apart from the acupuncture method, efforts are made to minimize the placebo effect, including limiting interactions between therapists and participants, adding objective outcome measurements [83, 84]. In this systematic review, the outcomes included vertebrobasilar blood flow velocity. As for the objective outcomes, they can be less affected by performance bias. Secondly, our analysis found moderate heterogeneity across studies, which might be caused by different intervention types, acupuncture types, and acupoint selection. To decrease the heterogeneity, we conducted subgroup analyses according to acupuncture type and acupuncture course. Significantly, the inconsistent therapies reminded us of a lack of standard procedure. Hence, the exploration of different acupoints and acupuncture's does-effectare still needed to help build standard treatment guidelines. Thirdly, for clinical efficacy evaluation, the Clinical Effective Rate was calculated based on the Guiding Principles for Clinical Study of New Chinese Medicines and Criteria for the Diagnosis and Treatment of Traditional Chinese Medicine Syndrome, which lacked a worldwide representative standard. To form a comprehensive evaluation, this study got the vertebrobasilar blood flow velocity included as the outcome index.

## 5. Conclusions

This study suggests that acupuncture therapy improves the blood flow velocity of vertebrobasilar arteries and Clinical Effective Rate in patients with posterior circulation infarction vertigo. Subgroup analysis reveals that longer acupuncture intervention course and higher stimulation are more effective in improving vertebrobasilar blood flow velocity. However, double-blind, sham-controlled trials with large sample sizes are required to further verify acupuncture's therapeutic efficacy in posterior circulation infarction vertigo.

## Figures and Tables

**Table 1:** Characteristics of the included studies.

Author, year	Study design	Sample size	Participants		Intervention			Main Outcomes
			Average age (years)	Gender (male/female)	Treatment group	Control group	Course (days)	
Dong et al., 2020 [36]	RCT	90	T: 51.4 ± 9.4; C: 52.6 ± 7.7	T: 25/20; C: 27/18	Manual acupuncture Flunarizine hydrochloride	Flunarizine hydrochloride	21	①②③④⑦
Li et al., 2020 [37]	RCT	80	T: 61.53 ± 4.53; C: 62.13 ± 4.57	T: 22/18; C: 21/19	Manual acupuncture Conventional treatment	Conventional treatment	20	⑥⑧
Zhang et al., 2020 [38]	RCT	60	—	—	Manual acupuncture Conventional treatment	Flunarizine hydrochloride Conventional treatment	10	①②③④⑤
Deng et al., 2019 [39]	RCT	60	T: 62.17 ± 9.76; C:62.1 ± 10.17	T: 11/19; C: 12/18	Manual acupuncture Conventional treatment	Conventional treatment	10	①⑤⑧
Dang, 2018 [40]	RCT	69	T:62.26 ± 20.23; C: 61.78 ± 21.57	T: 14/25; C: 11/19	Manual acupuncture Flunarizine hydrochloride Betastatin	Flunarizine hydrochloride Betastatin	10	①⑦
Zhang et al., 2017 [41]	RCT	60	T: 62.40 ± 9.7; C: 61.30 ± 10.1	T: 11/19; C: 10/20	Manual acupuncture Conventional treatment	Flunarizine hydrochloride Conventional treatment	7	①②③④
Zhou et al., 2017 [42]	RCT	60	T: 57.9 ± 12.8; C: 60.4 ± 12.7	T: 23/17; C: 11/9	Manual acupuncture Conventional treatment	Vinpocetine injection Conventional treatment	14	①
Chai, 2016 [43]	RCT	92	T: 56.7 ± 6.1; C: 57.3 ± 6.9	T: 27/19; C: 25/21	Manual acupuncture Flunarizine hydrochloride Conventional treatment	Flunarizine hydrochloride Conventional treatment	20	①②③④⑤⑦
Chen et al., 2016 [44]	RCT	70	All: 49.3 ± 6.5	T: 19/16; C: 17/18	Electroacupuncture Conventional treatment	Conventional treatment	14	①
Geng, 2016 [45]	RCT	70	T: 59.3; C: 61	T: 20/15; C: 17/18	Manual acupuncture Betastatin Danhong injection	Betastatin Danhong injection	14	①②③④
Zhang et al., 2014 [46]	RCT	78	T: 56.86; C: 57.02	T: 17/22; C: 15/24	Electroacupuncture Conventional treatment	Flunarizine hydrochloride Conventional treatment	10	①②③④
Zhou et al., 2014 [47]	RCT	60	All: 54.0 ± 3.8	T: 18/17; C: 15/10	Manual acupuncture Pushen capsule	Pushen capsule	15	①
Cheng et al., 2013 [48]	RCT	72	T: 51.81 ± 5.63; C: 52.17 ± 6.07	T: 13/22; C: 12/25	Manual acupuncture Conventional treatment	Nimodipine Conventional treatment	20	①⑤
Qian et al., 2013 [49]	RCT	98	T: 55.74 ± 5.36; C: 58.63 ± 5.75	T: 26/23; C: 24/25	Manual acupuncture Conventional treatment	Conventional treatment	14	①②③④
Wang, 2013 [50]	RCT	80	T: 45–75; C: 48–77	T: 23/17; C: 20/20	Manual acupuncture Alprostadil injection	Alprostadil injection	14	①
Li et al., 2012 [51]	RCT	112	T: 60.2 ± 8.2; C: 59.1 ± 7.2	T: 29/30; C: 26/27	Manual acupuncture Conventional treatment	Conventional treatment	20	①
Liu et al., 2012 [52]	RCT	71	T: 60.46 ± 8.47; C: 59.83 ± 9.36	T: 18/19; C: 14/20	Electroacupuncture	Flunarizine hydrochloride	42	①②③④
Ma and Zhang, 2012 [53]	RCT	120	T: 60.0 ± 3.4; C: 62.0 ± 2.8	T: 21/39; C: 18/42	Manual acupuncture Gastrodin injection Conventional treatment	Gastrodin injection Conventional treatment	10	①②③④
Luo, 2010 [54]	RCT	61	T: 63.74 ± 16.75; C: 62.98 ± 19.42	T: 20/11; C: 18/12	Manual acupuncture Conventional treatment	Conventional treatment	18	①
Gao and Zhao, 2009 [55]	RCT	78	T: 60.49 ± 4.87; C: 59.89 ± 5.06	T: 24/18; C: 20/16	Manual acupuncture Conventional treatment	Flunarizine hydrochloride Conventional treatment	15	①

T: treatment group; C: control group; ① Clinical Effective Rate; ② blood flow velocity of basilar artery; ③ blood flow velocity of left vertebral artery; ④ blood flow velocity of right vertebral artery; ⑤ Traditional Chinese Medicine Vertigo Symptom Scale; ⑥ Dizziness Handicap Inventory; ⑦ Symptom Scale of Traditional Chinese Medicine; ⑧ Activity of Daily Living.

**Table 2:** Characteristics of acupuncture.

Study	Main Acupoints	Acupuncture type	Reinforcing and reducing	Retention time
Dong et al., 2020 [36]	EX-HN3 (Yintang), PC 6 (Neiguan), SP 6 (Sanyinjiao), DU 20 (Baihui),GB 20 (Fengchi), GB 12 (Wangu), BL 10 (Tianzhu)	MA^1^	Mild supplementing and reducing	30 min
Li et al., 2020 [37]	GB 20 (Fengchi), DU 20 (Baihui), DU 21 (Qianding), DU 19 (Houding, EX-HN5 (Taiyang)	MA	Mild supplementing and reducing	30 min
Zhang et al., 2020 [38]	GB 20 (Fengchi), DU 16 (Fengfu), DU 20 (Baihui), GB 12 (Wangu), BL 10 (Tianzhu)	MA	Mild supplementing and reducing	30 min
Deng et al., 2019 [39]	GB 20 (Fengchi), GB 12 (Wangu), BL 10 (Tianzhu), DU 20 (Baihui), LR 3 (Taichong), GB 39 (Xuanzhong), ST 8 (Touwei), PC 6 (Neiguan), EX-HN3 (Yintang), ST 36 (Zusanli), RN6 (Qihai), RN 4 (Guanyuan)	MA	Mild supplementing and reducing	30 min
Dang et al., 2018 [40]	DU 20 (Baihui), GB 20 (Fengchi), BL 2 (Cuanzhu)	MA	—	—
Zhang et al., 2017 [41]	Faxuan, Xuanhuan	MA	Mild supplementing and reducing	30 min
Zhou and Gao, 2017 [42]	GB 20 (Fengchi)	MA	Mild supplementing and reducing	30 min
Chai, 2016 [43]	GB 20 (Fengchi), SI 15 (Jianzhongshu)	MA	Mild supplementing and reducing	20 min
Chen et al., 2016 [44]	GB 20 (Fengchi), Gongxue, EX-HN14 (Yiming)	MA + EA^2^	Mild supplementing and reducing, sparse-dense wave	30 min
Geng et al., 2016 [45]	GB 20 (Fengchi), DU 20 (Baihui), PC 6 (Neiguan)	MA	Mild supplementing and reducing	30 min
Tian 2014	RN 17 (Danzhong), RN 12 (Zhongwan), RN6 (Qihai), ST 36 (Zusanli), SP 10 (Xuehai), SJ 5 (Waiguan)	MA	Mild supplementing and reducing	—
Zhang et al., 2014 [46]	GB 20 (Fengchi), Gongxue	MA + EA	Mild supplementing and reducing, sparse-dense wave with 2 Hz	30 min
Zhou et al., 2014 [47]	Faxuan, Longji, Xiangling	MA	—	—
Cheng et al., 2013 [48]	GB 20 (Fengchi), DU 20 (Baihui), DU 14 (Dazhui), DU 16 (Fengfu), DU 23 (Shangxing), BL 17 (Geshu), Gongxue, Ashi	MA	Mild supplementing and reducing	—
Qian et al., 2013 [49]	GB 20 (Fengchi), DU 20 (Baihui), PC 6 (Neiguan), LR 3 (Taichong), BL 18 (Ganshu), BL 23 (Shenshu)	MA	Mild supplementing and reducing	30 min
Wang, 2013 [50]	GB 20 (Fengchi), GB 12 (Wangu), BL 10 (Tianzhu), DU 20 (Baihui), EX-HN1 (Sishencong)	MA	Mild supplementing and reducing	30 min
Li et al., 2012 [51]	LR 3 (Taichong)	MA	Mild supplementing and reducing	20 min
Liu et al., 2012 [52]	DU 20 (Baihui), EX-B2 (Cervical Jiaji)	MA + EA	Sparse-dense wave	30 min
Ma and Zhang, 2012 [53]	GB 20 (Fengchi), DU 20 (Baihui), BL 10 (Tianzhu), EX-HN1 (Sishencong)	MA	Mild supplementing and reducing	—
Luo, 2010 [54]	DU 20 (Baihui), DU 26 (Shuigou), DU 14 (Dazhui), RN 24 (Chengjiang), RN 4 (Guanyuan), RN 6 (Qihai)	MA	—	30 min
Gao and Zhao, 2009 [55]	GB 20 (Fengchi), DU 16 (Fengfu), EX-HN14 (Yiming), Gongxue	MA	—	30 min

^1^Manual acupuncture; ^2^electroacupuncture.

**Table 3:** Risk of bias of the included randomized controlled trials.

Study	Randomization process	Deviations from intended interventions	Missing outcome data	Measurement of the outcome	Selection of the reported result	Overall bias
Dong et al., 2020 [36]	Low	Low	Low	Low	Some concerns	Low
Li et al., 2020 [37]	Low	Low	Low	Low	Some concerns	Low
Zhang et al., 2020 [38]	Low	Low	Low	Low	Some concerns	Low
Deng et al., 2019 [39]	Low	Low	Low	Some concerns	Some concerns	Some concerns
Dang et al., 2018 [40]	Low	Low	Low	Some concerns	Some concerns	Some concerns
Zhang et al., 2017 [41]	Low	Low	High	Low	Some concerns	High
Zhou and Gao, 2017 [42]	Low	Low	Low	Low	Some concerns	Some concerns
Chai, 2016 [43]	Low	Low	Low	Low	Some concerns	Some concerns
Chen et al., 2016 [44]	Low	Low	Low	Low	Some concerns	Some concerns
Geng et al., 2016 [45]	Some concerns	Low	Low	Low	Some concerns	Some concerns
Zhang et al., 2014 [46]	Some concerns	Low	Low	Low	Some concerns	Some concerns
Zhou et al., 2014 [47]	Low	Low	Low	Some concerns	Some concerns	Some concerns
Cheng et al., 2013 [48]	Some concerns	Low	Low	Low	Some concerns	Low
Qian, 2013 [49]	Low	Low	Low	Low	Some concerns	Low
Wang, 2013 [50]	Some concerns	Low	Low	High	Some concerns	High
Liu et al., 2012 [52]	Some concerns	Low	Low	Low	Some concerns	Low
Li et al., 2012 [51]	Some concerns	Low	Low	Some concerns	Some concerns	Some concerns
Ma and Zhang, 2012 [53]	Some concerns	Low	Low	Low	Some concerns	Some concerns
Luo, 2010 [54]	Some concerns	Low	High	Some concerns	Some concerns	High
Gao and Zhao, 2009 [55]	Some concerns	Low	Low	Some concerns	Some concerns	Some concerns
Kappa	0.688	1	0.688	1	1	1

**Table 4:** Egger's test.

Std_Eff	Coef.	Std. Err.	*t*	*P* > |*t*|	(95% CI)	
Slope	0.0040179	0.0928593	0.04	0.966	−0.2003641	0.2083999
Bias	1.602403	0.9917507	1.62	0.134	−0.580425	3.785232

## Data Availability

Data generation and analysis information are available from the corresponding author on reasonable request.

## Supplementary Materials

Supplementary Materials Supplementary Table S1 provides the search terms and search strategy of this study. Supplementary Table S2 shows the number of articles in each database. PRISMA 2020 Checklist contains the checklist items of a systematic review, including the title, abstract, introduction, methods, results, and discussion.
