# Supplementary material for: Cerebral Blood Flow Velocity Modulation and Clinical Efficacy of Acupuncture for Posterior Circulation Infarction Vertigo: A Systematic Review and Meta-Analysis
Source: Evid Based Complement Alternat Med. 2022 Jun 28;2022:3740856. doi: 10.1155/2022/3740856 (PMC9256413; doi:10.1155/2022/3740856)
Supplement: Supplementary Materials — Supplementary Table S1 provides the search terms and search strategy of this study. Supplementary Table S2 shows the number of articles in each database. PRISMA 2020 Checklist contains the checklist items of a systematic review, including the title, abstract, introduction, methods, results, and discussion. [file 3740856.f1.zip › 3740856.f1/Supplemental Table S1. Search strategy.docx]

Supplemental Table S1 Search strategy

| No. | Search strategy |
| --- | --- |
| #1 | "vertigo"[MeSH Terms] OR "vertigo"[All Fields] OR "vertigos"[All Fields] OR "vertigoes"[All Fields] OR ("vertigo"[MeSH Terms] OR "vertigo"[All Fields] OR ("central"[All Fields] AND "origin"[All Fields] AND "vertigo"[All Fields]) OR "central origin vertigo"[All Fields]) OR ("vertigo"[MeSH Terms] OR "vertigo"[All Fields] OR ("brain"[All Fields] AND "stem"[All Fields] AND "vertigo"[All Fields])) OR ("dizziness"[MeSH Terms] OR "dizziness"[All Fields] OR "dizzy"[All Fields] OR "vertigo"[MeSH Terms] OR "vertigo"[All Fields]) |
| #2 | "brain infarction"[MeSH Terms] OR ("brain"[All Fields] AND "infarction"[All Fields]) OR "brain infarction"[All Fields] OR ("posterior"[All Fields] AND "circulation"[All Fields] AND "brain"[All Fields] AND "infarction"[All Fields]) OR "posterior circulation brain infarction"[All Fields] OR (("posterior"[All Fields] OR "posteriors"[All Fields]) AND ("blood circulation"[MeSH Terms] OR ("blood"[All Fields] AND "circulation"[All Fields]) OR "blood circulation"[All Fields] OR "circulation"[All Fields] OR "circulations"[All Fields] OR "circulate"[All Fields] OR "circulated"[All Fields] OR "circulates"[All Fields] OR "circulating"[All Fields]) AND ("infarctation"[All Fields] OR "infarcted"[All Fields] OR "infarctic"[All Fields] OR "infarcting"[All Fields] OR "infarction"[MeSH Terms] OR "infarction"[All Fields] OR "infarct"[All Fields] OR "infarctions"[All Fields] OR "infarcts"[All Fields] OR "infarctive"[All Fields])) OR (("posterior"[All Fields] OR "posteriors"[All Fields]) AND ("blood circulation"[MeSH Terms] OR ("blood"[All Fields] AND "circulation"[All Fields]) OR "blood circulation"[All Fields] OR "circulation"[All Fields] OR "circulations"[All Fields] OR "circulate"[All Fields] OR "circulated"[All Fields] OR "circulates"[All Fields] OR "circulating"[All Fields]) AND ("stroke"[MeSH Terms] OR "stroke"[All Fields] OR "strokes"[All Fields] OR "stroke s"[All Fields])) OR (("posterior"[All Fields] OR "posteriors"[All Fields]) AND ("blood circulation"[MeSH Terms] OR ("blood"[All Fields] AND "circulation"[All Fields]) OR "blood circulation"[All Fields] OR "circulation"[All Fields] OR "circulations"[All Fields] OR "circulate"[All Fields] OR "circulated"[All Fields] OR "circulates"[All Fields] OR "circulating"[All Fields]) AND ("ischaemics"[All Fields] OR "ischemia"[MeSH Terms] OR "ischemia"[All Fields] OR "ischaemic"[All Fields] OR "ischemic"[All Fields] OR "ischemical"[All Fields] OR "ischemically"[All Fields] OR "ischemics"[All Fields] OR "ischemized"[All Fields])) OR ("vertebro basilar ischaemia"[All Fields] OR "vertebrobasilar insufficiency"[MeSH Terms] OR ("vertebrobasilar"[All Fields] AND "insufficiency"[All Fields]) OR "vertebrobasilar insufficiency"[All Fields] OR ("vertebro"[All Fields] AND "basilar"[All Fields] AND "ischemia"[All Fields]) OR "vertebro basilar ischemia"[All Fields]) OR ("vertebrobasilar insufficiency"[MeSH Terms] OR ("vertebrobasilar"[All Fields] AND "insufficiency"[All Fields]) OR "vertebrobasilar insufficiency"[All Fields]) |
| #3 | "acupunctural"[All Fields] OR "acupuncture"[MeSH Terms] OR "acupuncture"[All Fields] OR "acupuncture therapy"[MeSH Terms] OR ("acupuncture"[All Fields] AND "therapy"[All Fields]) OR "acupuncture therapy"[All Fields] OR "acupuncture s"[All Fields] OR "acupunctured"[All Fields] OR "acupunctures"[All Fields] OR "acupuncturing"[All Fields] OR ("electroacupuncture"[MeSH Terms] OR "electroacupuncture"[All Fields] OR "electroacupuncturing"[All Fields]) OR ("needle s"[All Fields] OR "needled"[All Fields] OR "needles"[MeSH Terms] OR "needles"[All Fields] OR "needle"[All Fields] OR "needling"[All Fields] OR "needlings"[All Fields]) OR ("needle s"[All Fields] OR "needled"[All Fields] OR "needles"[MeSH Terms] OR "needles"[All Fields] OR "needle"[All Fields] OR "needling"[All Fields] OR "needlings"[All Fields]) |
| #4 | "randomized controlled trial"[Publication Type] OR "randomized controlled trials as topic"[MeSH Terms] OR "randomized controlled trial"[All Fields] OR "randomised controlled trial"[All Fields] OR ("Controlled"[All Fields] AND ("clinical trials as topic"[MeSH Terms] OR ("clinical"[All Fields] AND "trials"[All Fields] AND "topic"[All Fields]) OR "clinical trials as topic"[All Fields] OR "trial"[All Fields] OR "trial s"[All Fields] OR "trialed"[All Fields] OR "trialing"[All Fields] OR "trials"[All Fields])) OR ("clinical trial"[Publication Type] OR "clinical trials as topic"[MeSH Terms] OR "clinical trial"[All Fields]) |
| #5 | #1 AND #2 AND #3 AND #4 |
