# Supplementary material for: Cerebral Blood Flow Velocity Modulation and Clinical Efficacy of Acupuncture for Posterior Circulation Infarction Vertigo: A Systematic Review and Meta-Analysis
Source: Evid Based Complement Alternat Med. 2022 Jun 28;2022:3740856. doi: 10.1155/2022/3740856 (PMC9256413; doi:10.1155/2022/3740856)
Supplement: Supplementary Materials — Supplementary Table S1 provides the search terms and search strategy of this study. Supplementary Table S2 shows the number of articles in each database. PRISMA 2020 Checklist contains the checklist items of a systematic review, including the title, abstract, introduction, methods, results, and discussion. [file 3740856.f1.zip › 3740856.f1/Supplemental Table S2. Number of articles in each database.docx]

| Database | Number of articles searched from each database | Number of articles extracted from each database | Total Number of articles included into meta-analysis |
| --- | --- | --- | --- |
| PubMed | 22 | 2 | 20 |
| EMBASE | 42 | 0 |  |
| Cochrane Library | 38 | 0 |  |
| Web of Science | 13 | 0 |  |
| Chinese Biomedical Literature Database | 91 | 3 |  |
| Chinese National Knowledge Infrastructure | 344 | 9 |  |
| Wanfang Database | 283 | 4 |  |
| VIP | 163 | 2 |  |

Supplemental Table S2. Number of articles in each database
